# The potential global distribution of the papaya mealybug, *Paracoccus marginatus*, a polyphagous pest

**DOI:** 10.1002/ps.6151

**Published:** 2020-11-04

**Authors:** Elizabeth A Finch, Tim Beale, Mani Chellappan, Georg Goergen, Basana Gowda Gadratagi, Mohammed Abul Monjur Khan, Abdul Rehman, Ivan Rwomushana, Arup Kumar Sarma, Kris AG Wyckhuys, Darren J Kriticos

**Affiliations:** ^1^ CABI, Bakeham Lane, Egham Surrey UK; ^2^ CABI, Nosworthy Way Wallingford UK; ^3^ Kerala Agricultural University Thrissur India; ^4^ International Institute of Tropical Agriculture (IITA) Cotonou Benin; ^5^ Division of Crop Protection ICAR – National Rice Research Institute Cuttack India; ^6^ Department of Entomology Bangladesh Agricultural University Mymensingh Bangladesh; ^7^ CABI, Shamsabad Rawalpindi Pakistan; ^8^ CABI Nairobi Kenya; ^9^ Assam Agricultural University Jorhat India; ^10^ Chrysalis Consulting Hanoi Vietnam; ^11^ Fujian Agriculture and Forestry University Fuzhou China; ^12^ University of Queensland Brisbane Queensland Australia; ^13^ CSIRO Canberra Australian Capital Territory Australia

**Keywords:** bioclimatic model, climatic suitability, process modelling, CLIMEX, niche model

## Abstract

**BACKGROUND:**

The papaya mealybug, *Paracoccus marginatus*, is a highly polyphagous invasive pest that affects more than 200 plants, many of which are of economic importance. We modelled the potential distribution of *P. marginatus* using CLIMEX, a process‐oriented, climate‐based niche model. We combined this model with spatial data on irrigation and cropping patterns to increase the real‐world applicability of the model.

**RESULTS:**

The resulting model agreed with known distribution points for this pest and with broad areas where *P. marginatus* has been reported, but for which no GPS data were available. Our model highlights the potential expansion of *P. marginatus* into novel areas in Central and East Africa, as well as further expansion in Central America and Asia, as these areas are highly climatically suitable, and have large expanses of suitable crop hosts. It also highlights areas, such as the central and eastern states of the USA as well as the western provinces of China, that are suitable for seasonal invasions of *P. marginatus*.

**CONCLUSION:**

Our results offer refined resolution on areas with high potential for invasion by *P. marginatus*. © 2020 Society of Chemical Industry

## Graphical abstract

In the present study we present, for the first time, the potential global distribution of *Paracoccus marginatus* in order to highlight areas which are suitable for invasion.

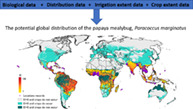

## 1 INTRODUCTION

The papaya mealybug, *Paracoccus marginatus* Williams and Granara de Willink (Hemiptera: Pseudococcidae) is a polyphagous insect pest that has a host range of over 200 plants,^1^ including economically important crops such as *Citrus* spp. L. (citrus), *Carica papaya* L. (papaya), *Manihot esculenta* (cassava) and *Persea americana* P. Mill. (avocado).^2^ Infestation by *P. marginatus* is highly detrimental to the host plant, and can result in crop losses of up to 91%,^3^ although it is important to note the crop losses are highly crop‐dependent; for example, yield loss in cassava, papaya and mulberry ranges from 10% to 60%.^4^ Crop losses due to *P. marginatus* can have severe economic impacts – one study found that infestations of papaya orchards in Bangladesh led to an average economic loss of approximately US$700 per hectare per year (range from US$413–1268).^5^ Another study in Ghana showed that infestations in a papaya orchards led to a 65% yield loss, which resulted in reduced export earnings and the loss of jobs for 1700 people.^6^ Thus, controlling the spread of *P. marginatus* has clear agricultural, economic and social benefits.

While female *P. marginatus* have no wings and are only capable of crawling short distances during early development instars, there are several other pathways by which *P. marginatus* are dispersed. Individuals have been transported up to several kilometers by wind^7^ and transportation *via* irrigation channels has also been shown.^8^ Further, they can be dispersed passively by other organisms, such as by crows and bats,^9^ as well as actively by certain ant species which farm the mealybugs for honeydew.^10^ Human mediated transport is key for their long‐distance dispersal, with transport of infested plant material a key risk.^8^ This is especially true given the wide range of economically important plant hosts which are exported around the world.

*Paracoccus marginatus* has spread rapidly over the last three decades. Thought to be native to Mexico and Central America,^11^ it was first recorded outside of this range in 1994 when it was found in the Caribbean.^12^ By 1998, it had spread to Florida, USA^11^, ^13^ and subsequently it was also found in Guam in 2002,^14^ in the Republic of Palau in 2003,^15^ and in Hawaii in 2004.^16^ It first appeared in Asia in 2008 when it was reported in Indonesia, southern India and Sri Lanka.^17^, ^18^ By 2009, it had also been found in Malaysia, Thailand, Lao PDR, Vietnam and Cambodia,^6^, ^17^, ^19^ spreading to Taiwan by 2011^20^ and to China by 2014.^21^ By late 2009, *P. marginatus* was found for the first time on the African continent in Ghana.^22^ In 2010, it was observed in Benin^6^ and by 2015, it was found in Tanzania and Mozambique.^21^, ^23^, ^24^ Most recently, this pest has been recorded in Israel,^25^ Gabon and Kenya.^3^

This pest has been so successful due to its quick development and prolific reproductive capacity.^26^, ^27^ Fortunately, the spread has been contained in many areas due to the introduction of biocontrol such as *Acerophagus papayae* and *Anagyrus loecki*.^4^, ^15^, ^28^ However, *Paracoccus marginatus* has the potential to spread to new areas and rapidly reach high numbers unless suitable phytosanitary or control methods are implemented. Hence, information about this pest's potential distribution is important as it can highlight key areas susceptible to invasion, giving an early warning to decision makers, allowing them to put into place phytosanitary measures to prevent or slow the invasion of the pest into their jurisdiction. While there have been previous attempts to model the potential distribution of *P. marginatus*, this has only been done at a local scale in Kenya,^29^ and as such a global view of the potential distribution is much needed. In this paper, we fit a semi‐mechanistic CLIMEX model for *P. marginatus* to estimate climatically suitable locations for this pest. Using this, alongside information on the areas where host crops are grown and where irrigation is used, we identified areas which could be invaded by *P. marginatus*.

## 2 MATERIAL AND METHODS

### 2.1 Current global occurrence records of *P. marginatus*

Global distribution points for *P. marginatus* were pooled from several publications,^5^, ^6^, ^9^, ^11^, ^14^, ^15^, ^16^, ^17^, ^18^, ^30^, ^31^, ^32^, ^33^, ^34^, ^35^ as well as a minority of unpublished sources. For papers with location information but without coordinate data, we geo‐referenced the points based on the place names provided (data available in online repository).^36^ We cleaned the data, removing points that were outside of country boundaries and also removed one observation which occurred on the border of USA and Canada, which we believe to be an individual which was intercepted during transportation of goods across the border. In total, 1094 records for *P. marginatus were collected*. To make data visualisation and manipulation easier, we thinned out the data using a grid with cell diameter of 10 arc minutes which resulted in a working total of 537 records.

### 2.2 Crop data

To determine the potential risk to agriculture, we used spatial data on cropping areas for a number of economically important crop hosts of *P. marginatus*, including avocado, bean, cashew, cassava, cherry, citrus, cocoa, coconut, cotton, cowpea, eggplant, maize, mango, okra, papaya, pea, pepper, pigeonpea, pineapple, potato, pumpkin, rubber, sunflower, sweet potato and tomato. This data was obtained from EARTHSTAT.^37^

### 2.3 CLIMEX modelling

CLIMEX^38^, ^39^ is a process‐oriented, climate‐based niche model that has been used widely to model the potential distribution of many invasive species.^40^, ^41^, ^42^ In order to assess the potential for a species to persist and grow in any given area (represented by the Ecoclimatic Index, EI), CLIMEX integrates an annual growth index (GI_A_) and stress indices (SI). The GI_A_ represents the potential for a population to grow, and combines the organism's response to temperature, soil moisture and, where relevant, day‐lengths. SI represents temperature and moisture stresses which potentially limit a species' geographical distribution. The EI is scaled from 0–100, where 0 is unsuitable for the survival of that species and 100 is ideal conditions. An EI score of 100 is very rarely seen and generally, EI > 30 represents very favorable climactic conditions for the given species.^38^ The projections of GI_A,_ SI and EI are created using (i) specific climatic parameters which are derived from information on the species' response to climate variables, and (ii) the known distribution of the species.^38^, ^39^, ^43^

### 2.4 Meteorological data and irrigation

We used the CliMond CM10 World (1975H V1.1) climate dataset to fit models under a natural rainfall scenario.^44^ This global dataset consists of 30‐year averages centred on 1975 for daily minimum and maximum temperatures, monthly rainfall totals and relative humidity (at 09:00 and 15:00 h), all at a spatial resolution of 10 arc minutes. Additionally, to account for the potential effects of irrigation we applied a top‐up irrigation scenario of 2.5 mm day^−1^ throughout the year. Two model scenarios were run in CLIMEX using the parameters described below; one model assumed that all areas were irrigated with up to 2.5 mm of water each day if less than 2.5 mm of rainfall was received that day, and the other model assumed no artificial irrigation. Irrigated areas were identified from Siebert *et al*.^45^ and used alongside the results from the two models to create a composite map of suitability for *P. marginatus*; for each 10′ cell, the irrigation model result was used in irrigated areas, and the non‐irrigated model result was used in non‐irrigated areas.

### 2.5 CLIMEX parameter fitting

#### 2.5.1 *Growth indices*

##### 2.5.1.1 Moisture index

*Paracoccus marginatus* is dependent on fresh plant material to survive, however prolonged exposure to abiotic stress, such as drought, often results in the weakening of a plant's defence system, making them more susceptible to pests. Because of this, the lower soil moisture threshold (SM0) was set at 0.1, which is roughly equivalent to the permanent wilting point for plants with moderate rooting depth. The lower optimum soil moisture level (SM1) was set slightly higher at 0.2 to suit the regions where *P. marginatus* records are found. While *P. marginatus* is present in many tropical and sub‐tropical regions, various reports suggest that population growth still occurs at a limited rate during the rainy season, but becomes explosive in the dry season if not controlled.^46^, ^47^ Accordingly, an upper optimum soil moisture level was set at 0.9, which is below the level of soil saturation, and the upper soil moisture threshold was set at 2 to allow persistence during the rainy season (Table 1).

**Table 1:** CLIMEX parameter values for *Paracoccus marginatus* modelling

Parameters	Description	Value	Unit
Moisture			
SM0	Lower soil moisture threshold	0.1	*
SM1	Lower optimum soil moisture	0.2	*
SM2	Upper optimum soil moisture	0.9	*
SM3	Upper soil moisture threshold	2	*
Temperature			
DV0	Lower temperature threshold	13	°C
DV1	Lower optimum temperature	27	°C
DV2	Upper optimum temperature	32	°C
DV3	Upper temperature threshold	38	°C
Cold stress			
TTCS	Cold stress temperature threshold	13	°C
THCS	Temperature threshold stress accumulation rate	−0.001	Week^−1^
Heat Stress			
TTHS	Heat stress temperature threshold	38	°C
THHS	Temperature threshold stress accumulation rate	0.001	Week^−1^
Dry stress			
SMDS	Soil moisture dry stress threshold	0.1	*
HDS	Stress accumulation rate	−0.001	Week^−1^
Wet stress			
SMWS	Soil moisture wet stress threshold	2	*
HWS	Stress accumulation rate	0.01	Week^−1^
Threshold heat sum			
PDD	Number of degree‐days above DV0 needed to complete one generation	300	°C days

^*^ Values without units are dimensionless indices of soil moisture.

##### 2.5.1.2 Temperature index

A study of *P. marginatus* by Amarasekare *et al*.^48^ estimated that the optimum and maximum temperature thresholds for adult males is 28.7 and 31.9 °C and for adult females is 28.4 and 32.1 °C, respectively. However, as these thresholds were based on experiments which were conducted under nearly constant temperatures and did not consider daily fluctuations, we concluded that in the natural environment, *P. marginatus* may be able to develop and survive at higher and lower temperatures than observed in this study. Indeed, when conducting a study in Mursidabad, India between August 2013 and July 2014, Lalitha *et al*.^49^ found the highest incidence of *P. marginatus* was in May 2014 which indicates there must have been growth of immature stages earlier in the year. Mean generation time has been found to range between 11–16.5 days^50^ and so immature stage growth must have occurred during April and May 2014 during which time the maximum daily temperature often exceeded 40 °C. Assuming that population growth took place in cooler parts of the day, we set the upper temperature threshold (DV3) at 38 °C. We set the upper optimum temperature (DV2) at 32 °C as this was a good fit to the known distribution of *P. marginatus* (Table 1).

Lalitha *et al*.^49^ reported a decline in populations of *P. marginatus* in West Bengal between November and March. This corresponded with mean monthly temperatures of between 18 and 22 °C. Given that the population was able to persist during these months, although presumably with much‐reduced reproduction, this indicates these temperatures lie somewhere between the lower temperature threshold (DV0) and the lower optimal temperature (DV1). Further, Lalitha *et al*.^49^ showed that once the mean monthly temperature increased to 27 °C, there was an increase in population in the next month suggesting that this temperature is within the optimal temperature conditions. Accordingly, we set the lower optimum temperature (DV1) to 27 °C (Table 1). Two studies have looked at the cumulative minimum development threshold of *P. marginatus*, one on potato (*Solanum tuberosum* L.), which found the threshold to be 13.9 °C in females and 14.5 °C in males^48^ and one on parthenium (*Parthenium hysterophorus* L.) which found the threshold to be 13 °C in females and 10 °C in males.^51^ We set DV0 to 13 °C, which fit well the known distribution of *P. marginatus* (Table 1).

Male and female *P. marginatus* have been estimated to require 303.0 and 294.1°‐days (DD), respectively, to complete their development.^48^ As such, we set the degree‐degree days per generation (PDD) to 300°‐degree days (Table 1).

#### 2.5.2 *Stresses*

##### 2.5.2.1 Heat and Cold stress

The heat stress temperature threshold (TTHS) and the cold stress temperature threshold (TTCS) were set at 38 and 13 °C, respectively for reasons explained above. Stress accumulations for TTHS and TTCS were set to 0.001 week^−1^ and − 0.001 week^−1^, respectively. These parameter values were well fitted to the known pest distribution.

##### 2.5.2.2 Dry stress

The dry stress threshold moisture level (SMDS) was set to 0.1, and dry stress accumulation (HDS) at a rate of −0.001 week^−1^.

##### 2.5.2.3 Wet stress

Heavy rain has been shown to negatively affect populations of *P. marginatus*.^46^ Thus, the wet stress parameters (SMWS) was set to 2 and the stress accumulation rate (HWS) was set to 0.01 week^−1^. These parameter values showed a good match with the known distribution of *P. marginatus*, especially in southern India.

#### 2.5.3 *Modelling uncertainty*

We conducted a sensitivity analysis to ascertain which parameters had the greatest influence over the model results. In these analyses, the values for all parameters for the fitted model were raised and lowered to see the effect on the results of the model.^38^ Parameters which, when altered, had a bigger effect on the model results were said to be sensitive. These analyses were only performed for the natural rainfall scenario.

## 3 RESULTS

**Figure 1:**
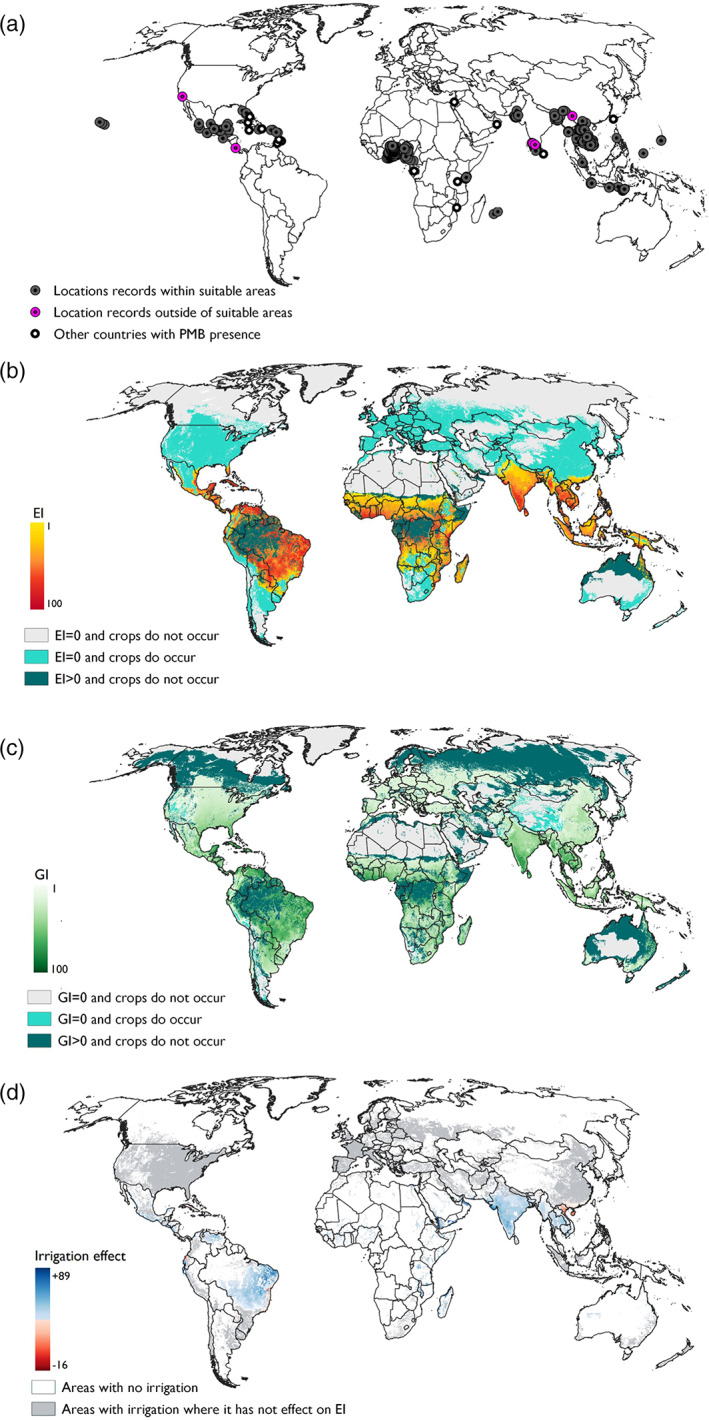
(a) *Paracoccus marginatus* distribution points used for building the model. Grey points represent locations which were within areas modelled as climatically suitable, whereas the six pink points represent locations which were outside of areas modelled as climatically suitable. Black dots represent the central points of countries where *P. marginatus* has been reported but for which precise locational information was unavailable. (b) Modelled global climate suitability for *P. marginatus* to persist as a permanent population when taking into consideration areas of irrigation and harvested areas of host plants (c) Modelled global climate suitability for *P. marginatus* to have positive growth in harvested areas of host plants under an irrigation scenario regardless of the potential to persist as a permanent population. (d) The effect of the irrigation scenario on climate suitability. Blue areas represent areas where the irrigation increased climate suitability, whereas red areas represent areas where irrigation decreased climate suitability.

The results from the model fit well the known distribution of *P. marginatus*, with 96.5% of distribution points falling in areas which were modelled as being environmental suitable (Fig. 1(a)). There were 19 distribution points that fell in areas modelled as being climatically unsuitable. However, 13 of these were just outside the spatial coverage of the environmental data, suggesting this was a spatial data precision problem. This left six outliers, three in southern India, one in southern China, one in Costa Rica and one in the USA, all of which were in areas modelled as having a positive growth index, suggesting some seasonal population growth is possible.

As well as describing the known distribution of *P. marginatus*, the model highlighted the potential for *P. marginatus* to extend beyond its current known distribution into other countries such as the Democratic Republic of Congo, Zambia, Brazil and Colombia. Given the high cover of crops favored by *P. marginatus* in these areas, the potential for invasion appears high.

### 3.1 The Americas

**Figure 2:**
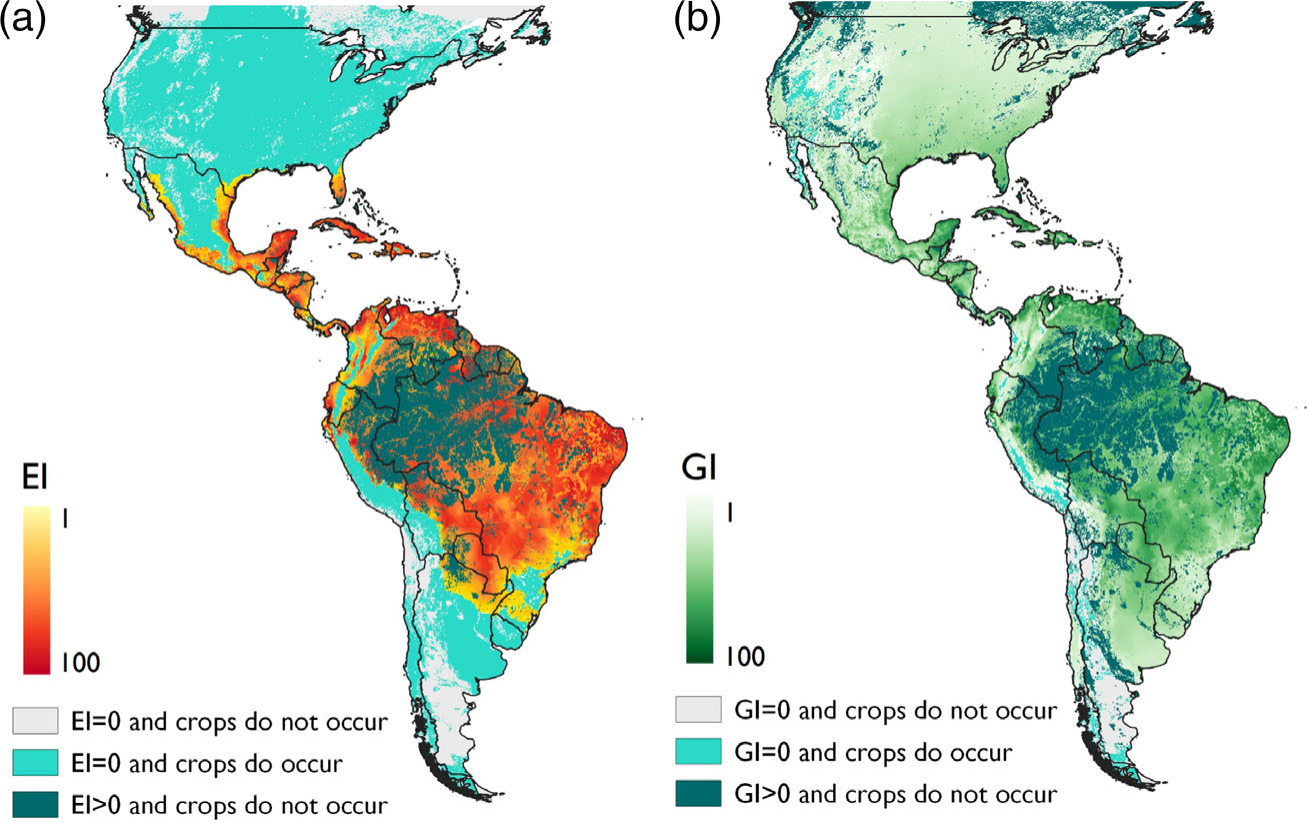
Modelled climate suitability of the Americas for *Paracoccus marginatus* (a) to persist as a permanent population, when taking into consideration areas of irrigation and harvested areas of host plants and (b) to have positive growth in harvested areas of host plants under an irrigation scenario regardless of the potential to persist as a permanent population.

Overall there was good fit between the modelled and known distribution of *P. marginatus* in its suspected native range of Mexico and Central America, with all points except one lying within areas categorized as climatically suitable (Fig. 2(a)).

El Salvador, Honduras, Nicaragua and Panama are outside of the current known distribution of *P. marginatus* but are modelled as having a high climate suitability. There is a possible that the pest is present in these areas and that it is simply not being recognized and/or reported, but if this is not true and *P. marginatus* is truly absent, the high climate suitability of these areas, along with their proximity to countries with recorded presence of *P. marginatus*, and the abundance of suitable crop hosts, suggests that their potential of being invaded is high.

While *P. marginatus* has been reported in French Guiana,^52^ there have been no official reports in any other country in South America. The CLIMEX model indicates high levels of suitability in most of South America ranging from Baranquilla in the north of Colombia, to the north tip of Uruguay. The mountainous regions of Colombia and Ecuador are not suitable due to excessive cold stress, nor is the eastern side of Peru and Bolivia or large swathes of Argentina except for an area in the north‐east. With the exception of the Amazon rainforest area in Brazil, the distribution of suitable crop hosts largely coincides with the areas modelled as highly suitable for *P. marginatus*, making them suitable for invasion.

The most southern tips of Texas and Florida in the USA have the potential for invasion as environmental conditions are highly suitable here, and there is an abundance of potential crop hosts. This fits well with the known distribution of *P. marginatus* in Florida – though these populations are now under successful control as a result of the release of four endoparasitoid wasp species: *Acerophagus papayae*, *Anagyrus loecki*, *Anagyrus californicus*, and *Acerophagus* (=*Pseudaphycus)* sp.^53^, ^54^ Cold stress is the main cause of unsuitability for establishment across the rest of the USA, however positive growth indices in California, as well as areas along the Pacific coastline and in the central and eastern states, suggests the potential for recurrent seasonal invasions of *P. marginatus* in these areas is quite high (Fig. 2(b)) if the four release endoparasitoid wasp species have not expanded into these areas.

### 3.2 Africa

**Figure 3:**
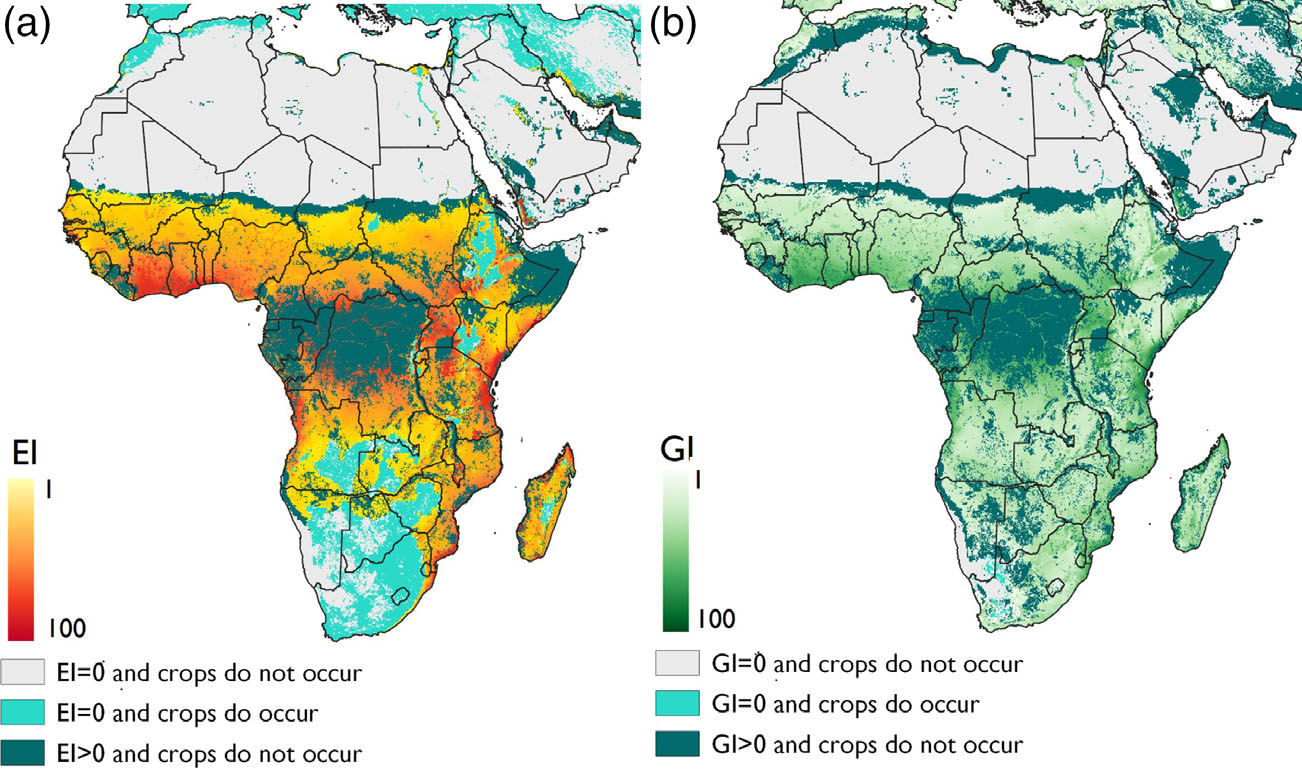
Modelled climate suitability of Africa for *Paracoccus marginatus* (a) to persist as a permanent population, when taking into consideration areas of irrigation and harvested areas of host plants and (b) to have positive growth in harvested areas of host plants under an irrigation scenario regardless of the potential to persist as a permanent population.

In Africa, the potential distribution of *P. marginatus* extends well beyond its known distribution, with most countries under the Sahel region having favorable climatic conditions for this pest. These areas largely coincide with the distribution of suitable crop hosts, making them highly suitable areas for invasion by *P. marginatus*. Areas of high suitability which did not coincide with suitable crop hosts were found in the north and central areas of the Democratic Republic of Congo, south Cameroon, The Republic of Congo, northern Somalia and east Ethiopia (Fig. 3(a)).

### 3.3 Asia

**Figure 4:**
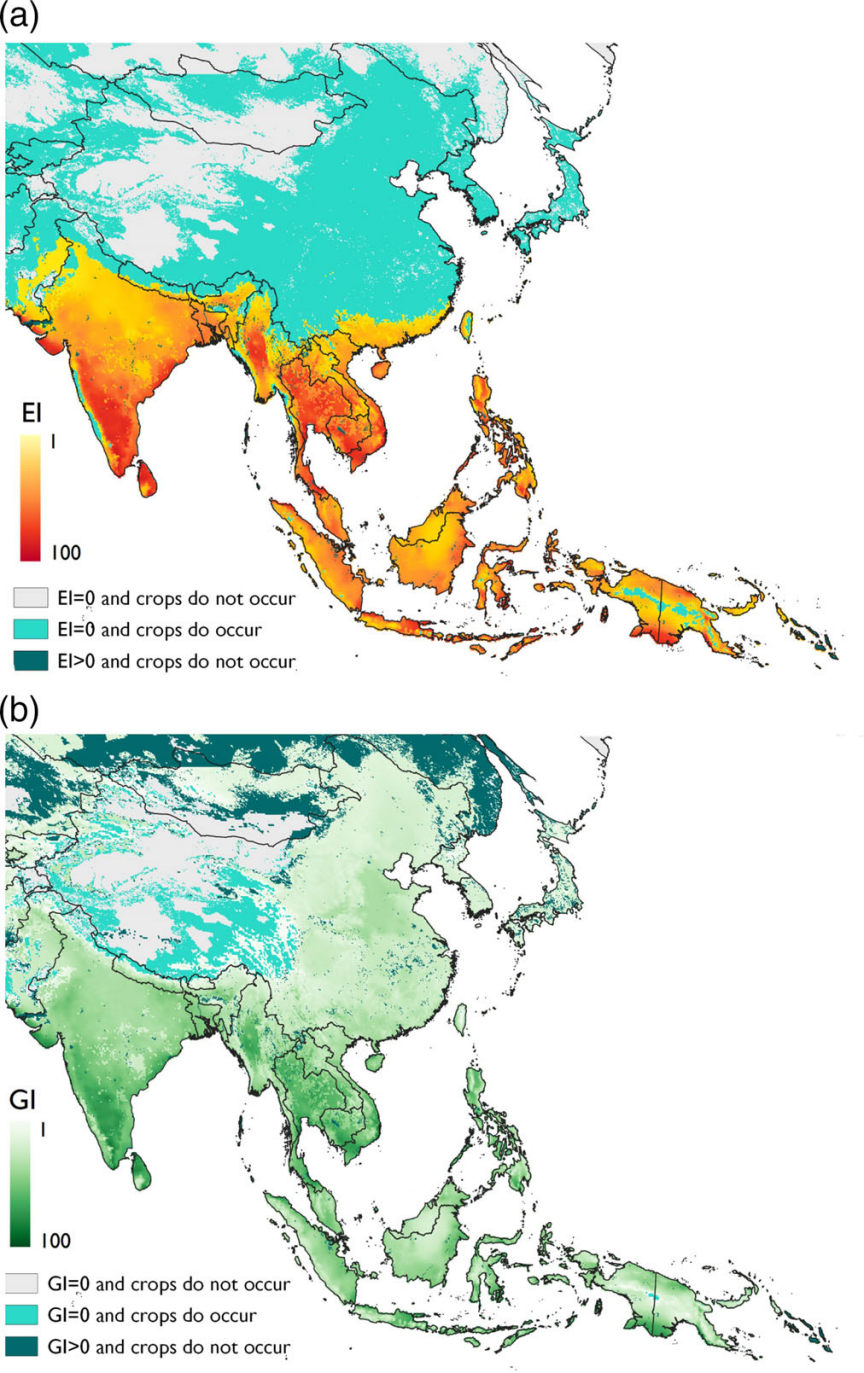
Modelled climate suitability of Asia for *Paracoccus marginatus* (a) to persist as a permanent population, when taking into consideration areas of irrigation and harvested areas of host plants and (b) to have positive growth in harvested areas of host plants under an irrigation scenario regardless of the potential to persist as a permanent population.

Suitable crop hosts of *P. marginatus* are distributed across large parts of Asia from Afghanistan in the east to Indonesia in the west. This overlaps with large areas modelled as highly suitable, specifically India, Southeast Asia and the southern regions of the Guangxi and Guangdong provinces of southern China, highlighting the potential for further widespread distribution of *P. marginatus* in these areas (Fig. 4(a)). The pest is unlikely to spread to most areas within the north‐western Chinese provinces of Tibet, XinJiang and Qinghai as the model suggests that *P. marginatus* populations would experience high levels of cold stress in these areas. There are, however, other areas in south‐eastern and north‐eastern China which are generally modelled as climatically unsuitable for *P. marginatus*, but do have positive growth indices, thus suggesting the potential of seasonal range expansion (Fig. 4(b)).

### 3.4 Australasia

Australia showed the largest divergence between areas of suitability and host plants. North Australia was largely modelled as suitable, while crop growth was mostly found in southern Australia (Fig. 1(b)). There was however, a small amount of fragmented land along the north‐eastern side of Queensland, from the very northern tip of Queensland to Bundaberg, where crop growth and climatic suitability did coincide.

### 3.5 Europe

Levels of cold stress were too high over the majority of Europe to be suitable for *P. marginatus* establishment. However, the model did indicate very small areas of land surrounding Seville in Spain and around Sicily in Italy that were climatically suitable (Fig. 1(b)). Further, positive growth indices did occur over the rest of mainland Europe and this largely intersected with areas of suitable crop types (Fig. 1(c)).

### 3.6 Effect of irrigation

The biggest effect of irrigation was seen in Brazil (specifically the central and eastern parts of the country) and in Asia (specifically India and across Southeast Asia), which all saw a large increase in their environmental suitability in locations where irrigation is practiced (Fig. 1(d)). Increased environmental suitability in the northern states of India corresponds with published observations of *P. marginatus* in Rajasthan^28^ and Gujarat.^55^ The south of China and the north of Vietnam saw their environmental suitability decrease in areas were irrigation is practiced, but this negative effect of irrigation was more localized and much less profound than the increase in environmental suitability seen in other areas (Fig. 1(d)).

### 3.7 Parameter sensitivity and model uncertainty

The cold stress temperature threshold (TTCS), cold stress temperature rate (THCS) and the limiting low moisture (SM0) were the most sensitive parameters as they had a 3.7%, 1.73% and 1.45% effect, respectively, on the modelled suitability range (Table 2). The sensitivity analysis for cold stress temperature was conducted over a range from 12–14 °C. While the value used in the model provides a good fit with the known distribution of *P. marginatus*, field studies conducted in West Bengal which were used to set the parameter value, suggest that the actual value does lie somewhere in the range tested by the sensitivity analysis.^49^ Further, we feel confident in the chosen value for cold stress temperature rate as it fits well with the known distribution of *P. marginatus*. The sensitivity analysis for the limiting low moisture parameter was conducted over a range from 0.0–0.2. *Paracoccus marginatus* is a plant pest, and as such is highly unlikely to grow in conditions that would be impossible for the growth of its plant host (less than permanent wilting point, SM0 < 0.1). However, when a plant is under drought stress (roughly at SM0 = 0.1), they have increased pest susceptibility. We are therefore confident in our value of 0.1 for SM0 as it represents the limiting conditions that a plant can survive in. All other parameters had a less than 1% effect on modelled range (Table 2).

**Table 2:** CLIMEX parameter sensitivity values for *Paracoccus marginatus* parameters, as applied to the CM10 1975H V1.1 global dataset under a natural rainfall scenario

Parameter	Mnemonic	Parameter values			Range change (%)	EI change	Core distribution change (%)	Growth variables			Stress variables		
		Low	Default	High				MI change	TI change	GI change	CS change	DS change	HS change
Cold Stress Temperature Threshold	TTCS	12	13	14	3.73	4.77	1.27	0	0	0	17.72	0	0
Cold Stress Temperature Rate	THCS	−0.0012	−0.001	−0.0008	1.73	1.87	0.20	0	0	0	7.16	0	0
Limiting low moisture	SM0	0	0.1	0.2	1.45	4.34	0	18.0	0	7.73	0	0	0
Lower optimal moisture	SM1	0.1	0.2	0.3	0.52	3.75	0	15.7	0	6.66	0	0	0
Limiting high temperature	DV3	37	38	39	0.20	1.21	0	0	3.34	1.39	0	0	0
Dry Stress Threshold	SMDS	0	0.1	0.2	0.20	0.66	4.77	0	0	0	0	25.62	0
Heat Stress Temperature Threshold	TTHS	37	38	39	0.10	0.69	0.28	0	0	0	0	0	7.64
Upper optimal temperature	DV2	31	32	33	0.08	2.50	0	0	4.94	2.74	0	0	0
Heat Stress Temperature Rate	THHS	0.0008	0.001	0.0012	0.06	0.20	0	0	0	0	0	0	2.86
Wet Stress Threshold	SMWS	1.9	2	2.1	0.06	0.90	0.70	0	0	0	0	0	0
Limiting low temperature	DV0	12	13	14	0.04	1.15	0	0	3.74	2.60	0	0	0
Wet Stress Rate	HWS	0.008	0.01	0.012	0.02	0.40	0.08	0	0	0	0	0	0
Upper optimal moisture	SM2	0.8	0.9	1	0	2.40	0	5.55	0	2.61	0	0	0
Limiting high moisture	SM3	1.9	2	2.1	0	2.16	0	3.29	0	2.27	0	0	0
Lower optimal temperature	DV1	26	27	28	0	2.57	0	0	5.63	3.33	0	0	0
Dry Stress Rate	HDS	−0.0012	−0.001	−0.0008	0.02	0.17	0.58	0	0	0	0	3.50	0
Hot‐Dry Temperature Threshold	TTHD	0	0	1	0	0	0	0	0	0	0	0	0
Hot‐Dry Moisture Threshold	MTHD	0	0	0.1	0	0	0	0	0	0	0	0	0
Degree‐days per Generation	PDD	240	300	360	0	0	0	0	0	0	0	0	0

## 4 DISCUSSION AND CONCLUSIONS

*Paracoccus marginatus* is an invasive pest with a high economic cost and the ability to spread rapidly and infest multiple types of crops. In order to better control the existing spread and prevent further invasions into new areas, knowledge of local risks is required. Here we have pooled data from 34 countries and overseas territories to model the potential distribution of this pest and highlight areas which have the greatest potential for invasion.

There is substantial potential for the expansion of *P. marginatus* into cropping areas in central and eastern Africa and South America. Further the model indicates that there is substantial potential for expansion in Central America and into Asia, where suitable host crops are highly abundant.

### 4.1 Fit of model

The high level of agreement (96.5%) between the known current distribution of *P. marginatus* and our modelled suitable prediction demonstrates the reliability of our model.

In our model, only six distribution points were located outside of areas modelled as suitable. Three of these points were found in the southern India state of Kerala and were unsuitable due to wet stress. Kerala is one of the states that receives the highest levels of rainfall in India, which has been shown to negatively affect *P. marginatus*.^46^ A positive growth index in this area suggests that populations of *P. marginatus* can locally persist during the dry season, when this data point was collected,^32^ but may be greatly reduced in the rainy season. The points in southern China and in the USA were in areas unsuitable due to cold stress. Both areas however, have positive growth indices suggesting that populations of *P. marginatus* could persist there in the warmer months of the year, and that perhaps these records represent transient populations.

Our model also indicates favorable environmental suitability in other countries where *P. marginatus* have been recorded but which were not used to fit the model, including: Barbados,^21^ Cuba,^56^ Dominican Republic,^2^ Gabon,^3^ Israel,^25^ Mozambique,^21^ Oman,^21^ Saint Lucia,^21^ Sri Lanka,^18^ Taiwan^20^ and Tanzania.^3^ Further, sensitivity analysis showed that there were no highly sensitive, poorly understood parameters that might impact upon the reliability of our model predictions.

### 4.2 Cropping extent

To get a better understanding of the risk of *P. marginatus* to agriculture, we examined the pest's modelled potential distribution alongside crop distribution. *Paracoccus marginatus* is highly polyphagous, with over 200 recorded host plants^1^: however, the crop distribution used in our maps covers just 25 crops for which distribution data were available. The extent of the crop distribution is, therefore, likely to be a conservative estimate of suitable crop cover. This is an important consideration as the potential distribution of *P. marginatus* could expand into areas that are currently shown as being climatically suitable but having no suitable crop growth. This is particularly true where *P. marginatus* could spread into vegetation which is a non‐economically important plant host, of which *P. marginatus* has many.^1^ This should also be a consideration when identifying pathways of invasion from one area to another.

### 4.3 Model limitations

Here we have presented results that bring together climatic data, pest physiological attributes, and crop range data to assess the potential of *P. marginatus* to invade large swathes of area globally. Sensitivity analysis suggested that the model was fairly robust to changes in the potential inaccuracies in the fitted parameter values. We were also able to corroborate our model results with real world observations, which aligned closely.

One consideration regarding the results of our model is that it does not account for areas where the climatic conditions are artificially changed other than by irrigation. For example, growth of crops within a greenhouse, could not be accounted for. This is particularly pertinent for countries that grow large quantities of food within greenhouses such as North America, where approximately 20% of the tomato crop is cultivated in greenhouses.^57^

It is also worth noting that a species' ability to survive in an area's environmental conditions is just one component that affects its range – its ability to reach a specific site is equally crucial.^58^ Female mealybugs are not able to fly, so long distance dispersal of this pest is largely dependent on humans through the movement of infested plants/fruits,^8^ as is suggested by the apparent absence of seasonal spread from known infestations.

### 4.4 Management strategies

Results from our model highlight areas that are climatically suitable for *P. marginatus*, and have a high potential for invasion. We recommend intensifying phytosanitary efforts in these areas, in particular regarding trade in live plants and plant parts imported from a country with an existing *P. marginatus* population. For example, in 2018, Mexico, where *P. marginatus* originated, exported US$12.10 billion worth of fruit and vegetables to the USA, US$84.47 million of edible vegetables to Venezuela and US$46.54 million worth of edible fruits to Spain.^59^ Spain and Venezuela currently are not known to have populations of *P. marginatus*, but are climatically suitable for resident (Venezuela) and potential seasonal populations (Spain). Given the rapid and vast spread of *P. marginatus* across the globe, the implementation of phytosanitary measures in, for example, Spain can generate benefits for neighboring European countries which have suitable climate for seasonal populations of *P. marginatus*. Thus, focusing phytosanitary efforts on vectors for spread from known areas affected by *P. marginatus* into climatically suitable regions, is key.

We have highlighted areas *e.g*., in Asia or Central and North America, which are climatically suitable for a further expansion of either resident or seasonal populations of *P. marginatus*. Over the past years, parasitic wasps, principally *Acerophagus papayae* and *Anagyrus loecki*, have been deployed within several of these regions and have effectively controlled invasive *P. marginatus* populations.^15^, ^60^, ^61^ However it is important to note that in recently invaded areas, where host populations are low, effective chemical control is likely to be the best management strategy. In areas where *P. marginatus* has become established and reached a high enough population density, however, use of parasitoids remains an effective potential control method. Further ecological niche modelling of these parasitoid species is recommended to anticipate their survival, fitness and ultimate biological control impact in areas into which *P. marginatus* could potentially expand and become established. Previous studies on other parasitoids have shown that they are absent from range‐margin populations of the host because these are the most thermally challenging areas.^62^ If this is also true for the parasitoids of *P. marginatus*, then they will be ineffective as biocontrol at the edge of the potential range of *P. marginatus*. Sustained monitoring and data sharing will thus be critical elements of a *P. marginatus* containment and mitigation strategy. Further, given the likelihood of anthropogenic long‐distance dispersal of *P. marginatus*, there will be a continued need for vigilance and scientifically‐guided and quick responses to emerging pest outbreaks or invasions.

## CONFLICT OF INTEREST

The authors declare that they have no conflict of interest.
